# Perceptions towards physical activity in adult lung transplant recipients with cystic fibrosis

**DOI:** 10.1371/journal.pone.0229296

**Published:** 2020-02-21

**Authors:** Martina Wietlisbach, Christian Benden, Angela Koutsokera, Kathleen Jahn, Paola M. Soccal, Thomas Radtke

**Affiliations:** 1 Zurich University of Applied Sciences, School of Health Professions, Institute of Physiotherapy, Winterthur, Switzerland; 2 Division of Pulmonology, University Hospital Zurich, Zurich, Switzerland; 3 Division of Pulmonology, Centre Hospitalier Universitaire Vaudois, Lausanne, Switzerland; 4 Clinic of Pulmonary Medicine and Pulmonary Cell Research, University Hospital of Basel, Basel, Switzerland; 5 Division of Pulmonary Diseases, Geneva University Hospitals, Geneva, Switzerland; 6 Division of Occupational and Environmental Medicine, Epidemiology, Biostatistics and Prevention Institute, University of Zurich and University Hospital Zurich, Zurich, Switzerland; Teesside University/Qatar Metabolic Institute, UNITED KINGDOM

## Abstract

**Background:**

Barriers and motives towards physical activity (PA) in lung transplant (LTx) recipients with cystic fibrosis (CF) are largely unknown. We aimed to explore perceptions towards PA in LTx recipients with CF to better understand individuals’ needs and preferences.

**Methods:**

Participants completed an online survey at two Swiss LTx and one follow-up shared care centre between June and December 2018.

**Results:**

One hundred and eleven individuals completed the survey (87.4% response rate). Overall, survey participants perceive PA as important for their daily life and health. Perceived motives of PA were *improving muscle strength*, *endurance* and *quality of life* (QoL), *to feel better*, *fun*, *to achieve personal goals* and having *more energy for everyday life*. *Fatigue* was the most common perceived barrier to PA and associated with poorer QoL (r = -0.43, *p*<0.001) and health status (r = -0.31, *p* = 0.001). Participants with lung allograft dysfunction (LAD, n = 20) reported lower habitual PA (*p* = 0.009) and health status (*p* = 0.011), and *rated shortness of breath*, *bad weather* and *concerns regarding lung rejection* higher than those without LAD (all *p*<0.05). When we asked how an optimal training programme should look like, the majority would prefer individual, non-supervised (60%), outdoor (77%), endurance training (90%), once or twice a week (47%) for 40–60 minutes (48%). Only a minority of patients (14%) would be willing to use exercise applications for their home-based training.

**Conclusions:**

LTx recipients with CF value PA as important for their health. People with CF should be encouraged individually by their multidisciplinary transplant team to implement PA in their daily life, potential barriers should be identified and addressed. Overall, knowledge on perceived barriers and motives for PA should be considered in the development of future patient-centred PA programmes.

## Introduction

Cystic fibrosis (CF) is the most common autosomal recessive disease in the European population caused by mutations in the gene encoding for the CF transmembrane conductance regulator protein. This life-limiting disease is characterised by progressive lung destruction and accumulation of cardiovascular disease risk factors that, in the majority of individuals, leads to respiratory failure [1]. The life expectancy of people living with CF has increased considerably over the last decades [2] with newborns expected to live into their fifth decade [3]. Lung transplantation (LTx) remains the ultimate treatment option to improve the health-related quality of life (QoL) and survival in carefully selected people with end-stage CF lung disease [4]. Post-transplant outcomes for people with CF are superior compared to other underlying lung diseases necessitating LTx [4]; however, stringent multidisciplinary care including medical treatment, psychological and physical therapy is required after LTx.

Physical activity (PA) and exercise training are established components in LTx care [5,6]. Despite substantial improvements in lung health following LTx, functional exercise capacity (e.g., six-minute walk test distance) and PA levels are still significantly reduced compared to healthy people [7,8]. Peripheral muscle weakness is very common in LTx recipients [9,10] and likely the result of long periods of immobilisation during intensive care units stays [9], lack of PA [11], immunosuppressive agents and allograft rejection [11,12]. Structured exercise training following LTx has been shown to improve PA, QoL and exercise capacity in LTx recipients [9,11,13]. In order to achieve a long-term benefit on physical fitness, LTx recipients should be instructed and motivated to engage in regular, individually adapted PA. However, motivational problems and barriers to PA are common in non-transplanted people with CF [14,15], but knowledge on people with CF who have undergone LTx has not been investigated.

Previous studies assessing solid organ transplant recipients have shown that common barriers to PA are physical limitations (e.g., lack of strength), insufficient energy, fear, presence of comorbidities, side effects from medications and lack of exercise guidelines [16,17]. Factors that increase the likelihood of performing PA are a high level of motivation to stay healthy, consequences of physical (in)activity, having goals and/or prioritising goals, social support from family and friends, physician recommendation and responsibility for the transplanted organ (among others) [16,17]. Of note, individual factors such as having a goal or prioritising a goal may be perceived as both–motive and barrier for PA–depending on whether a goal priorisation is linked to PA or any other aspect of life [16]. While, solid organ transplant recipients share many disease-related characteristics (e.g., peripheral muscle weakness, comorbidities), PA levels show large variations across different types of solid organ transplants [17]. Moreover, the younger group of LTx recipients with CF may differ in many aspects from other transplant recipients such as professional reintegration.

The aim of this study was to explore perceptions towards PA in LTx recipients with CF to better understand their needs and preferences for regular PA. Specifically, we were interested to assess motivations and barriers for PA and to elucidate whether the duration of living with the new organ (i.e., years after LTx) and the presence of lung allograft dysfunction (LAD) impacts on peoples’ perceptions towards PA. Finally, we wanted to learn how the ideal exercise programme to improve a person’s physical fitness after LTx would look like from the perspective of the individuals.

## Materials and methods

We conducted a cross-sectional online survey at the two Swiss Transplant Centres, Centre Universitaire Romand de Transplantation (CURT) and Zurich, and a follow-up Shared Care Centre in Basel, Switzerland between June 1^st^ 2018 and 31^st^ December 2018. People with CF who had undergone LTx in Switzerland (CURT, Zurich) were eligible for this study. To be invited for participation, the following criteria had to be fulfilled: i) age 15 years or older, ii) time after LTx being at least 6 months, iii) comprehension of German and/or French language and iv) verbal informed consent. None of the transplant donors were from a vulnerable population and all donors or next of kin provided written informed consent that was freely given.

Two members of the Study Team (MW and TR) visited all collaborating centres instructing centre staff with respect to the use of the tablet computer (Lenovo TB-X103F) and the online survey. For hygienic reasons, the tablet was covered with a screen protector to allow appropriate disinfection after each use. For individuals infected with specific pathogens (e.g., *Burkholderia cepacia complex*), the tablet was packed in a transparent film that was destroyed after each single use.

During the study period, centre staff contacted potentially eligible participants during their routine clinical visits, explained the purpose of the study and invited them to complete the online survey, after verbal consent has been obtained.

This study does not fall under the scope of the Human Research Act. The Ethical Committee of the Canton of Zurich confirmed with a clarification of responsibility that ethical approval was not necessary for this study (2018–00389). In accordance with the Human Research Act in Switzerland, young people aged 14 years and older are allowed to provide consent to participate in a research project, provided they are able to judge (e.g., do not suffer from a mental disability). Moreover, written informed consent (i.e., signature by participant or parent/caregiver) is not mandatory since our study included only an online questionnaire (i.e., project with minimal risks) and the answers are anonymous. We systematically obtained verbal/oral consent from all participants. Each study site investigator created a datasheet/list with potentially eligible patients in their center, contacted patients during their routine clinical visits, explained the purpose of the project and recorded the verbal consent status prior to participating in the study.

### Questionnaire on motives and barriers of physical activity

We developed a self-administered questionnaire with the support of a LTx physician, a physiotherapist, an exercise scientist and a nutrition specialist with particular experience in the development of assessment and measurement tools. The anonymised questionnaire was drafted with SoSci Survey [18] and contained questions on demographic, anthropometric, and medical characteristics as well as questions on motives (i.e., factors that increase the likelihood of participating in PA) and barriers (i.e., factors that hinder participation in PA) towards PA. The latter were pre-specified options given on a 0–6 Likert scale (0 = not at all relevant, 6 = highly relevant) including additional options for individual answers (i.e., free text). The questions on barriers and motives for PA were designed after considering various studies on this topic in chronic disease populations. Further, questions about the optimal training programme to improve physical fitness from the perspective of the participants was also integrated. The original study questionnaires (German and French) and a version translated in English are given in the online supplementary material (S1–S3 Appendices).

Prior to starting the survey, the online questionnaire was pilot-tested with five LTx recipients [2 females, median (IQR) age 34 (30, 37) years, median (IQR) time after LTx 4 (2, 4) years)] from the Transplant Centre in Zurich. The pilot was done to evaluate the comprehensibility of the questions and the instrument as a whole, and to estimate the time needed to complete the survey. The individuals’ feedback was implemented into the final version.

### Quality of life

The EuroQol (EQ-5D-5L), a generic, self-administered instrument was used to assess QoL [19]. The EQ-5D-5L is a valid health outcome instrument in CF [20] and has been applied in various LTx populations including CF [21,22]. The instrument comprises five health dimensions: mobility, self-care, usual activities, pain/discomfort and anxiety/depression. Each dimension has five response categories: no problems, slight problems, moderate problems, severe problems, and extreme problems. The five health states were converted into an index value according to the German value set and using the Crosswalk Index Value Calculator provided by the EuroQol group (https://euroqol.org). The EQ-5D-5L also includes a visual analogue scale (VAS) with marked intervals from 0 (worst imaginable health state) to 100 (best imaginable health state).

### Habitual physical activity

Habitual PA was assessed with the short version of the International Physical Activity Questionnaire (IPAQ-SF), a tool with reasonable measurement properties to assess population-based PA levels [23]. The IPAQ-SF consists of 7 items and records the total minutes spent on vigorous PA, moderate PA, walking, and sedentary behaviour over the past 7 days; PA volumes are standardised according to metabolic equivalents (MET’s) and expressed as MET minutes per week (MET min.week^-1^). Participants were considered to have met PA recommendations by the World Health Organisation [24] if they reported at least 150 min.week^-1^ of walking, moderate or vigorous PA.

### Statistical analysis

All statistical analyses were performed with the statistical software package SPSS (IBM Corporation 2017, Version 25.0). Data from the online survey was exported into an Excel spreadsheet (Microsoft Excel 2018, Version 16.16.8) prior to the import in SPSS. IPAQ questionnaire data was cleaned and outliers were excluded in accordance with the IPAQ guidelines for data analysis and processing. Descriptive data is presented as median (interquartile range) or n (%). Spearman rank correlations were used to assess relationships between outcome variables. The Mann-Whitney-U test and the chi-square test (categorical variables) were used for comparisons between the two groups of individuals with and without LAD. We used a general linear model to determine differences between motivations and barriers to PA between three groups of individuals categorised according to the years after LTx (<3 years, 3 to <10 years and ≥10 years). The latter group was defined as the reference group. For all comparisons, the level of statistical significance was set to p<0.05.

## Results

Among 129 eligible LTx recipients, 127 were invited to participate. Of those, 111 adults with a median (IRQ) age of 35 (29, 41) years completed the survey (80/81 in Zurich, 17/32 at CURT, 14/14 in Basel) given a response rate of 87.4%. Participant characteristics are shown in Table 1. The median (IQR) time to complete the online survey was 10.9 (8.7, 12.7) minutes.

**Table 1:** Participants’ characteristics.

Variables	All (n = 111)	LAD-free (n = 91)	LAD (n = 20)	*P*-value
Age, years	35 (29, 41)	34 (29, 41)	37 (31, 42)	0.264
Sex, n (% female)	53 (48)	44 (48)	9 (45)	0.787
Years since LTx	4.0 (2.0, 5.0)	3.0 (2.0, 5.0)	4.5 (3.3, 5.0)	**0.037**
Re-LTx, n (%)	2 (2)	1 (1)	1 (1)	0.237
BMI, kg.m^-2^	20.3 (18.4, 22.4)	20.7 (18.4, 21.5)	20.2 (18.5, 22.5)	0.794
**Employment status**				
Employment, n (%)	73 (66)	64 (70)	9 (45)	**0.031**
Workload, %	50 (40, 80)	50 (40, 80)	50 (30, 80)	0.555
Invalidity pension, n (%)	86 (78)	68 (75)	18 (90)	0.140
**Comorbidities**				
Heart disease, n (%)	4 (4)	3 (3)	1 (1)	0.713
Hypertension, n (%)	44 (40)	36 (32)	8 (40)	0.971
Diabetes, n (%)	80 (72)	64 (70)	16 (80)	0.385
Kidney disease, n (%)	15 (14)	10 (11)	5 (25)	0.099
Liver disease, n (%)	5 (5)	4 (4)	1 (5)	0.906
Cancer*, n (%)	8 (7)	6 (7)	2 (10)	0.595
Depression, n (%)	12 (11)	10 (11)	2 (10)	0.898
Osteoporosis, n (%)	41 (37)	34 (37)	7 (35)	0.844
Incontinence, n (%)	4 (4)	3 (3)	1 (5)	0.713
**Quality of life**				
EQ-5D-5L index value	0.85 (0.71, 1.00)	0.85 (0.74, 1.00)	0.80 (0.64, 0.90)	0.187
EQ-5D VAS (0–100 scale)	80 (70, 90)	80 (70, 90)	70 (49, 84)	**0.011**
**Physical activity**				
Intense PA pre-LTx (hours.week^-1^)	3 (1, 5)	3 (1, 5)	4 (2, 9)	0.119
Total METs (min.week^-1^)	2988 (1448, 5970)	3372 (1613, 6933)	1796 (371, 3067)	**0.009**
Sitting time (min.day^-1^)	300 (240, 420)	300 (240, 420)	330 (240, 525)	0.537

Data are median (interquartile range) or number (percent). BMI, body mass index; EQ-5D-5L, EuroQol five dimensions questionnaire; LAD, lung allograft dysfunction; LTx, lung transplantation; MET, metabolic equivalent; PA, physical activity [assessed with the Short-Form International Physical Activity Questionnaire (IPAQ-SF)], VAS, visual analogue scale.

*Other than cancer of the skin.

### Motivations and barriers for participation in physical activity

**Fig 1:**
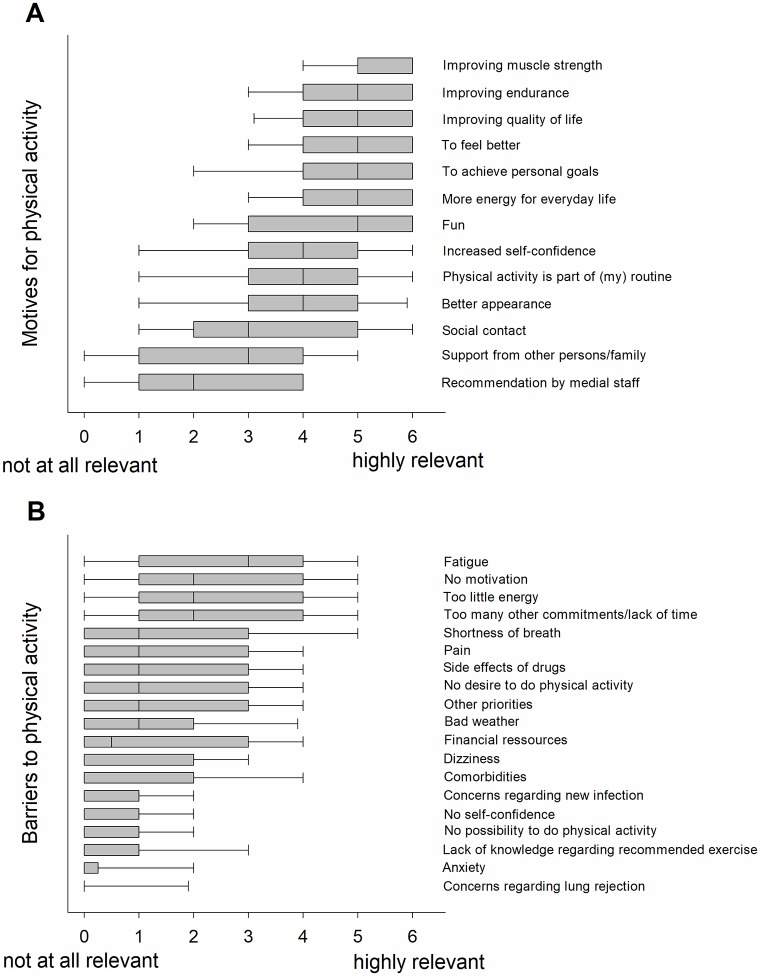
Motives (A) and barriers (B) to physical activity (PA) in lung transplant recipients with cystic fibrosis (CF). Boxplots indicate median values with interquartile ranges (25^th^ and 75^th^ percentiles) and error bars. Motives and barriers to PA are sorted (top to bottom) according to the highest median values (i.e., highest values on the 0–6 Likert scale).

The participants (n = 111) perceived PA as very important for their daily life [median 5, IQR (4, 5)] and health [median 5 IQR (5, 6)], see Fig 1. The most common motives were *improving muscle strength*, *endurance* and *QoL*, *to feel better*, *fun*, *to achieve personal goals*, and having *more energy for everyday life* (Fig 1A). *Fatigue* (i.e., feeling tired) was the most commonly perceived barrier for PA and was associated with poorer QoL (r = -0.43, *p*<0.001) and health status (r = -0.31, *p* = 0.001). Other reported barriers for PA were *no motivation*, *too little energy* and *too many other commitments/lack of time* (Fig 1B). Selected responses on barriers and motives towards PA from individual survey participants are given below:

“I have a dog that gets me out of doors, besides that I often lack energy to do anything else.”

“I consider sports activity to be an important therapy before AND after transplantation! Especially, for physical and psychological balance. After 3 days without sport I get dissatisfied and kind of depressed.”

“Endurance training is almost impossible, because there is always a physical disability which stops me.”

Additional, individual responses from survey participants are listed in the online supplements (S4 Appendix).

### Motivations and barriers for physical activity among participants with/without lung allograft dysfunction

The group of participants with LAD (n = 20, Table 1) rated barriers to PA higher than the group without LAD (n = 91). Between-group differences in median (IQR) values were found for *shortness of breath* [2.5 (0, 5) versus 0 (0, 2), *p* = 0.004], *concerns about lung rejection* [0 (0, 1.75) versus 0 (0, 0), *p* = 0.033] and *bad weather* [2 (1, 3) versus 1 (0, 2), *p* = 0.007]. There were no significant differences between groups regarding PA motives.

### Motivations and barriers of physical activity between groups categorised according to time after LTx

Characteristics of participants categorised in three different groups according to time after LTx (in years) are shown in Table 2. Participants who had received a LTx less than 3 years ago rated the motives *to improve QoL*, *to achieve personal goals*, having *more energy for everyday life*, *support from other persons/family* and *recommendation by medical staff* higher than those of the reference group (i.e., LTx ≥10 years ago), see Table 3. In addition, barriers to PA such as *concerns about lung rejection*, *anxiety*, *no self-confidence* and *financial resources* were also given higher importance compared to the reference group (Table 3). The only difference between those who were transplanted 3 to <10 years ago compared to the reference group, was found in the motive *to improve QoL*.

**Table 2:** Participants’ characteristics categorised according to time after lung transplantation (in years).

Variables	LTx < 3 years (n = 31)	LTx 3 to < 10 years (n = 46)	LTx ≥ 10 years (n = 34)	*p*-value
Age, years	30 (24, 35)	34 (29, 41)	40 (35, 49)	**<0.001**
Sex, n (% female)	14 (45)	22 (48)	22 (65)	0.212
Re-LTx, n (%)	0 (-)	2 (4)	0 (-)	0.237
BMI, kg.m^-2^	20.8 (18.2, 23.1)	20.0 (18.1, 21.8)	20.7 (18.7, 22.0)	0.634
LAD, n (%)	4 (13)	6 (13)	10 (29)	0.116
**Employment status**				
Employment, n (%)	19 (61)	30 (65)	24 (71)	0.729
Workload, %	50 (40, 75)	50 (30, 80)	50 (43, 95)	0.916
Invalidity pension, n (%)	23 (74)	37 (80)	26 (76)	0.802
**Comorbidities**				
Heart disease, n (%)	2 (6)	1 (2)	1 (3)	0.595
Hypertension, n (%)	7 (23)	16 (35)	21 (62)	**0.004**
Diabetes, n (%)	25 (81)	32 (70)	23 (68)	0.448
Kidney disease, n (%)	2 (6)	2 (4)	11 (32)	**0.001**
Liver disease, n (%)	0 (-)	2 (4)	3 (9)	0.230
Cancer*, n (%)	1 (3)	1 (2)	6 (18)	**0.018**
Depression, n (%)	1 (3)	6 (13)	5 (15)	0.269
Osteoporosis, n (%)	7 (23)	17 (37)	7 (21)	0.073
Incontinence, n (%)	2 (6)	1 (2)	1 (3)	0.595
**Quality of life**				
EQ-5D-5L index value	0.85 (0.74, 1.00)	0.86 (0.71, 1.00)	0.84 (0.71, 0.90)	0.987
EQ-5D VAS (0–100 scale)	83 (70, 91)	80 (70, 90)	73 (60, 80)	**0.041**
**Physical activity**				
Intense PA pre-LTx (hours.week^-1^)	4 (2, 10)	3 (1, 6)	2 (1, 4)	0.119
Total METs (min.week^-1^)	3078 (1448, 7902)	2998 (1403, 6197)	2739 (1346, 3943)	0.739
Sitting time (min.day^-1^)	300 (240, 420)	300 (240, 420)	360 (233, 435)	0.659

Data are median (interquartile range) or number (percent). BMI, body mass index; EQ-5D-5L, EuroQol five dimensions questionnaire; LAD, lung allograft dysfunction; LTx, lung transplantation; MET, metabolic equivalent; PA, physical activity [assessed with the Short-Form International Physical Activity Questionnaire (IPAQ-SF)], VAS, visual analogue scale.

*Other than cancer of the skin.

**Table 3:** Motivations and barriers to physical activity among lung transplant recipients with cystic fibrosis.

	LTx < 3 years *β* -coefficient (95% CI)	*p*-value	LTx 3 to < 10 years *β* -coefficient (95% CI)	*p*-value
**Facilitators**				
To feel better	0.00 (-0.62, 0.62)	1.000	-0.35 (-0.92, 0.21)	0.218
Increased self-confidence	0.32 (-0.54, 1.18)	0.462	0.24 (-0.55, 1.03)	0.544
Improving quality of life	0.63 (0.08, 1.17)	**0.024**	0.55 (0.06, 1.05)	**0.030**
Improving muscle strength	0.25 (-0.30, 0.79)	0.369	0.23 (-0.27, 0.72)	0.362
Improving endurance	0.24 (-0.29, 0.78)	0.367	0.21 (-0.28, 0.70)	0.388
Social contact	0.56 (-0.32, 1.45)	0.209	0.58 (-0.23, 1.39)	0.161
To achieve personal goals	1.00 (0.19, 1.80)	**0.016**	0.23 (-0.51, 0.97)	0.538
More energy for everyday life	0.99 (0.34, 1.64)	**0.003**	0.34 (-0.26, 0.93)	0.267
Fun	0.57 (-0.21, 1.35)	0.149	0.47 (-0.25, 1.18)	0.199
Physical activity is part of (my) routine	0.49 (-0.33, 1.31)	0.237	0.68 (-0.07, 1.43)	0.076
Support from other persons/family	1.80 (0.96, 2.64)	<0.001	0.38 (-0.39, 1.15)	0.327
Recommendation by medical staff	1.38 (0.64, 2.12)	**<0.001**	0.44 (-0.24, 1.12)	0.205
Better appearance	0.49 (-0.35, 1.32)	0.249	0.38 (-0.38, 1.14)	0.323
**Barriers**				
Dizziness	0.33 (-0.48, 1.13)	0.421	0.61 (-0.13, 1.34)	0.105
Shortness of breath	0.16 (-0.77, 1.08)	0.738	-0.42 (-1.26, 0.43)	0.329
Concerns regarding new infection	0.50 (-0.04, 1.03)	0.068	0.11 (-0.38, 0.60)	0.665
Concerns regarding lung rejection	0.71 (0.13, 1.28)	**0.017**	0.02 (-0.51, 0.54)	0.949
Anxiety	0.67 (0.17, 1.17)	**0.009**	0.14 (-0.31, 0.60)	0.537
No self-confidence	0.85 (0.28, 1.41)	**0.004**	-0.08 (-0.60, 0.44)	0.765
No motivation	0.33 (-0.58, 1.24)	0.469	-0.40 (-1.23, 0.43)	0.346
Fatigue	0.32 (-0.65, 1.30)	0.510	-0.10 (-0.99, 0.79)	0.826
Too many other commitments/lack of time	0.25 (-0.67, 1.16)	0.596	0.00 (-0.83, 0.84)	0.993
Too little energy	-0.38 (-1.34, 0.59)	0.442	-0.55 (-1.43, 0.33)	0.219
Side effects of drugs	-0.54 (-1.41, 0.33)	0.218	-0.46 (-1.25, 0.34)	0.255
Comorbidities	-0.44 (-1.27, 0.38)	0.292	-0.33 (-1.09, 0.43)	0.388
Pain	-0.19 (-1.09, 0.70)	0.671	-0.14 (-0.96, 0.68)	0.730
Financial resources	0.82 (-0.01, 1.64)	0.053	0.02 (-0.73, 0.78)	0.952
Bad weather	0.14 (-0.60, 0.88)	0.708	-0.29 (-0.96, 0.39)	0.403
No possibility to do physical activity	0.51 (-0.02, 1.04)	0.057	0.31 (-0.17, 0.79)	0.211
No desire to do physical activity	0.01 (-0.86, 0.88)	0.978	-0.17 (-0.97, 0.63)	0.670
Lack of knowledge regarding recommended exercise	-0.04 (-0.74, 0.66)	0.915	-0.21 (-0.85, 0.43)	0.522
Other priorities	-0.29 (-1.16, 0.58)	0.506	-0.48 (-1.28, 0.32)	0.234

Data are *β* -coefficients from general linear models with their 95% confidence intervals. LTx, lung transplantation. The group LTx ≥10 years was used as reference.

### Habitual physical activity

Habitual PA levels showed a large heterogeneity within the population sample. The median (IQR) IPAQ total activity score was 2988 (1448, 5970) MET min.week^-1^ and sitting time was 300 (240, 420) minutes.day^-1^, respectively. Among 104 individuals (49 females) with valid IPAQ questionnaire data, 63% achieved the PA recommendations. The group of participants with LAD had a lower activity score than those without LAD [1796 (371, 3067) versus 3372 (1613, 6933) MET min.week^-1^, *p* = 0.009].

### Quality of life

The median (IQR) EQ-5D index value was 0.85 (0.71, 1.0) and the patient-reported health status on the VAS was 80 (70, 90). Participants with LAD reported lower health status than those without LAD [70 (49.3, 83.8) versus 80 (70, 90) *p* = 0.011]. No differences were found in EQ-5D index among individuals with or without LAD [0.80 (0.64, 0.90) versus 0.85 (0.74, 1.00), *p* = 0.187], but those with LAD reported lower scores in the health dimensions mobility (*p* = 0.003), self-care (*p* = 0.016), and usual activities (*p* = 0.026), while the dimensions pain/discomfort (*p* = 0.878) and anxiety/depression (*p* = 0.256) were not different between groups.

### Optimal exercise training programme to improve physical fitness

Participants’ responses regarding the optimal training programme to improve physical fitness are given in Table 4. Among the pre-defined options, the majority of participants would prefer individual, non-supervised (60%), outdoor (77%), endurance training (90%), once or twice a week (47%) for 40–60 minutes (48%). Only a small proportion of participants would prefer to use exercise applications and/or DVD’s for their home-based training (14%) or would be interested to perform exercise training in a hospital or rehabilitation centre (11%). There were no differences in responses between individuals with LAD versus without LAD.

**Table 4:** Participants’ responses regarding the optimal exercise training program to improve their physical fitness (n = 111).

Specifications of exercise training	N (%)
**Type of training**	
Strength training	81 (73)
Endurance training	100 (90)
Balance training	37 (33)
No training at all	0 (0)
**Training supervision**	
Supervised group training	27 (24)
Individual, supervised training	43 (39)
Individual, unsupervised training	67 (60)
**Training venue**	
Home-based	53 (48)
Home-based with use of exercise-applications or CD/DVD	15 (14)
Outdoor	85 (77)
Sports club	33 (30)
Fitness centre	54 (49)
Institution (Hospital, Rehabilitation center, Physiotherapy)	12 (11)
**Training frequency**	
Daily	12 (11)
1–2 times per week	52 (47)
3–4 times per week	43 (39)
5–6 times per week	11 (10)
**Training duration**	
10–20 minutes per session	12 (11)
20–40 minutes per session	37 (33)
40–60 minutes per session	53 (48)
>60 minutes per session	19 (17)

Data are expressed as numbers (%).

All questionnaire data are available in the online supplements (S5 Appendix).

## Discussion

This is the first study investigating perceptions towards PA in adult LTx recipients with CF and aiming to explore participants’ opinions about the optimal exercise programme to improve physical fitness. We found that LTx recipients with CF value PA as highly important for their daily life and health. Motivations and barriers for PA appear to differ between groups of participants, depending on the time occurred since LTx as well as presence/absence of LAD.

In our survey, the most important perceived motives for PA were *improving muscle strength*, *endurance* and *QoL*, *to feel better*, *fun*, *to achieve personal goals* and having *more energy for everyday life*. *Fatigue*, *too little energy* and *too many other commitments/lack of time* were the most highly rated barriers for PA. Consistent with our observations, previous studies in solid organ transplant recipients [16,17] revealed that physical limitations, insufficient energy level and lack of muscle strength were important barriers (among others) for PA. In fact, 79% of our participants rated *improving muscle strength* with a score of 5 or 6 on a 0–6 Likert scale (0 = not at all relevant, 6 = highly relevant), indicating their motivation for muscle function improvement. This is not surprising because muscle weakness is common among LTx recipients [9,10] and is the main determinant of impaired exercise capacity [8] with potential consequences for daily life activities and QoL. Lack of muscle strength in LTx recipients is of multifactorial origin and the consequence of physical inactivity, immobilisation, and the detrimental impact of immunosuppressive drugs (among other factors) [12]. The dominant participant-reported barrier to PA in our study was *fatigue*, a subjective feeling of tiredness or exhaustion that is believed to be perpetuated by systemic, physical and psychological as well as behavioural factors [25]. Fatigue is highly prevalent in chronic respiratory diseases and associated with decreased physical and mental dimensions of health status [26]. Although, fatigue was not assessed rigorously using validated tools in this study (e.g., by considering both perceived and performance fatigability [27]), our observations extend previously reported relationships between fatigue and health status in chronic lung disease [26] to the population of LTx recipients with CF.

Interestingly, motivations and barriers to PA appear to differ depending on the time since LTx. The group of participants receiving a LTx less than 3 years ago rated *improvement in QoL*, *achievement of personal goals*, *support from other persons/family*, having *more energy for everyday life* and *recommendation by medical staff* higher than those living for ≥10 years with a LTx. On the other hand, the barriers *concerns regarding lung rejection*, *anxiety* and *no self-confidence* appear to hamper PA participation in this group of people. After LTx, people with CF are confronted with numerous disease-related tasks and need to acquire new skills to manage their life. Personal goal setting is an important process and represents an effective behaviour change technique [28]. A recent study in LTx recipients suggests that adherence to treatment is better early after LTx [29] and social support from family, friends and the medical transplant team is likely to be crucial to long-term outcomes. While previous work in solid organ transplant recipients identified physician recommendation as relevant factor supporting PA participation [17], our data suggest a gap in patient education and point towards the need to recommend regular exercise and PA for every individual, independent of the time after LTx. With respect to motives for PA, our observations may—on the other hand—simply reflect a shift in participants’ priorities with a focus towards maintenance rather than improvement of their current health status (e.g., with other disease-related aspects becoming more relevant). While habitual PA levels did not differ between the three different groups, those who underwent LTx ≥10 years ago had a higher prevalence of comorbidities including kidney disease and cancer, that may consequently affect their perception on long-term goals, goal setting and QoL. On the contrary, those who underwent LTx less than 3 years ago still appear to perceive barriers to PA participation that underline their lack of confidence in the new organ.

One important aim of our survey was to explore participants’ preferences regarding the optimal exercise-training programme to improve physical fitness. The majority of participants would prefer individual, non-supervised, outdoor endurance training once or twice a week for about 40–60 minutes. Only a minority (14%) are interested in using exercise applications and/or DVD’s for their home-based training. Although, fitness trackers and smartphone applications have become increasingly popular in exercise research (e.g. to improve long-term adherence) such an interventional approach will possibly have limited success on a larger scale in this population. Nevertheless, individual participants for whom technology-based applications may be helpful could be identified through patient-education and supported long-term. Taken together, the findings from this survey highlight the need for individualised PA counselling taking into account individual (e.g., barriers and motives), interpersonal (e.g., social support), and environmental (e.g., accessibility) factors to promote PA participation.

This study should be viewed in light of its strengths and limitations. With a participation rate of 87.4% our study is highly representative for the Swiss population of LTx recipients with CF, but our findings may not be perfectly transferable to other countries with different socioeconomic status and healthcare systems. Given the nature of the survey, our data rely on self-reporting that is prone to bias such as social desirability and/or recall bias. In this regard, the IPAQ-SF questionnaire has been reported to overestimate PA levels in comparison to objective measures of PA such as accelerometry [30]. Accelerometry data would have provided a more precise measure of individual PA levels. However, our main focus was on motivations and barriers for PA and we wanted to implement an easily applicable instrument to estimate population-based PA levels. Moreover, the group of individuals with LAD was small (n = 20), and therefore, some comparisons with the group of individuals without LAD may lack statistical power (e.g., QoL). Finally, we conducted a cross-sectional survey that does not allow to drawing conclusions about causal relationships. Longitudinal studies addressing changes in perceptions towards PA and exercise incorporating measures of physical and psychological dimensions of health would help to better understand the needs and preferences of people with CF who had undergone LTx and to establish patient-centered interventions.

## Conclusions

In conclusion, adult LTx recipients with CF value PA as important for their daily life and health, but barriers to PA such as perceived *fatigue* is associated with poorer QoL and health status. Motivations and barriers to PA appear to differ depending on the presence/absence of LAD and the time since LTx, highlighting the need for individualised PA counselling. Our findings may inform healthcare professionals to develop patient-centred PA programmes incorporating individuals’ preferences and needs.

## Supporting information

S1 Appendix Questionnaire on perceptions towards physical activity in adult lung transplant recipients with cystic fibrosis (German version). (DOCX)

S2 Appendix Questionnaire on perceptions towards physical activity in adult lung transplant recipients with cystic fibrosis (French version). (DOCX)

S3 Appendix Questionnaire on perceptions towards physical activity in adult lung transplant recipients with cystic fibrosis (English version). (DOCX)

S4 Appendix Individual responses of survey participants. (DOCX)

S5 Appendix (XLSX)
