# Supplementary material for: Perceptions towards physical activity in adult lung transplant recipients with cystic fibrosis
Source: PLoS One. 2020 Feb 21;15(2):e0229296. doi: 10.1371/journal.pone.0229296 (PMC7034849; doi:10.1371/journal.pone.0229296)
Supplement: S1 Appendix — (DOCX) [file pone.0229296.s001.docx]

**S1 Fragebogen zur Einstellung gegenüber körperlicher Aktivität bei Patienten mit Cystischer Fibrose nach Lungentransplantation**

*- Original German Version -*

**Sprache:**

□ Deutsch □ Französisch

Wir sind eine Forschungsgemeinschaft der Universität Zürich, des Universitäts Spitals Zürich sowie der Zürcher Hochschule für Angewandte Wissenschaften und daran interessiert, mehr über Ihre Einstellung zu körperlicher Aktivität zu erfahren. Dazu befragen wir gezielt Betroffene mit Cystischer Fibrose (CF) nach Lungentransplantation, die in den Zentren Basel, Genf, Lausanne und Zürich nachbetreut werden. Wir haben einen Fragebogen erarbeitet, der neben medizinischen Fragen hauptsächlich Fragen zu Ihrer Einstellung zu körperlicher Aktivität und Ihrer Lebensqualität beinhaltet. Dieser Fragebogen soll uns helfen, mehr Informationen über Ihre Bedürfnisse zu diesem Thema zu erfahren.

Für das Ausfüllen des **anonymen Fragebogens** benötigen Sie ca. **10-15 Minuten**.

**A: Allgemeine Fragen**

**A1: Geschlecht:** □ Weiblich □ Männlich

**A2: Alter: _____**

**A3: Familienstand:**

□ Kein/e Partner/in

□ Partner/in ausserhalb des Haushalts

□ Partner/in im Haushalt

□ Familie mit Kindern

□ Alleinerziehende/r

**A4: Grösse (in Metern):** ______ m (z.B. 1.68m)

**A5: Gewicht (in Kilogramm):** ______ kg (z.B. 59.5 kg)

**A6: Vor wie vielen Jahren hatten Sie die Lungentransplantation?**

□ weniger als (<) 1 Jahr

□ 1 bis < 3 Jahren

□ 3 bis < 5 Jahren

□ 5 bis < 10 Jahren

□ 10 oder mehr Jahren

**A7: Hatten Sie eine zweite Lungentransplantation (Re-Lungentransplantation)?**

□ Ja □ Nein

**A8: Haben Sie eine akute oder chronische Abstossung der Lunge?**

□ Ja □ Nein

**A9: Haben Sie Begleiterkrankungen / weitere Diagnosen?**

- *Mehrfachnennungen möglich -*

□ Nein

□ Herzerkrankung

□ Hoher Blutdruck

□ Diabetes („Zuckerkrankheit“)

□ Chronische Nierenerkrankung, Dialyse oder Nierentransplantation

□ Chronische Lebererkrankung oder Lebertransplantation

□ Hautkrebs

□ Krebs (ausser Hautkrebs)

□ Depression

□ Osteoporose

□ Inkontinenz

□ Andere: _________________________________________

**A10: Haben Sie nach der/den Lungentransplantation/en jedes Mal an einem Rehabilitationsprogramm teilgenommen?**

□ Ja □ Nein

**B: Fragen zur Ausbildung/Beruf**

**B1: Sind Sie berufstätig?**

□ Ja □ Nein → Falls „Nein“ weiter zu Frage B3.

**B2: Wie viel Stellenprozent arbeiten Sie?** ________%

**B3: Welche Ausbildung(en) haben Sie gemacht oder machen Sie?**

- *Mehrfachnennungen möglich -*

□ Universität / Fachhochschule / Pädagogische Hochschule / ETH

□ Eidg. Diplom (Meisterdiplom), Eidg. Fachausweis / Techniker- oder Fachschule /Höh. Fachschule, HTL, HWV

□ Fachmittelschule / Maturitätsschule / Berufsmaturität / Lehrerseminar

□ Berufslehre / Anlehre

□ Obligatorische Schule abgeschlossen

□ Obligatorische Schule nicht abgeschlossen

**B4: Erhalten Sie eine IV-Rente?**

□ Ja □ Nein → Falls „Nein“ weiter zu Frage C1.

**B5: Zu welchem Prozentsatz sind Sie IV-berechtigt?** __________ %

**C: Fragen zur körperlichen Aktivität**

**C1: Wie wichtig ist Ihnen körperliche Aktivität im Alltag?**

0 = überhaupt nicht wichtig – 6 = sehr wichtig

**C2: Wie oft waren Sie in einer durchschnittlichen Woche (7 Tage) vor der Transplantation körperlich aktiv, in Form von anstrengender körperlicher Aktivität?**

(Bitte beachten Sie die letzten 2 Jahre vor der Transplantation nicht). Beispiele von anstrengender körperlicher Aktivität: Aerobic, schnelles Fahrradfahren, schnelles Schwimmen, Spielsportarten, Joggen. Im Allgemeinen alle Aktivitäten, bei denen Sie schwitzen, sich der Herzschlag erhöht und Sie schnell atmen müssen.

ungefähr ____ Stunde(n) pro Woche

**C3: Wie wichtig schätzen Sie regelmässige körperliche Aktivität für Ihre Gesundheit ein?**

0 = überhaupt nicht wichtig – 6 = sehr wichtig

**C4: Was motiviert Sie persönlich, körperlich aktiv zu sein?**

0 = überhaupt nicht wichtig – 6 = sehr wichtig

□ Sich besser fühlen

□ Mehr Selbstvertrauen

□ Verbesserung der Lebensqualität

□ Verbesserung der Muskelkraft

□ Verbesserung der Ausdauer

□ Kontakt zu anderen Personen

□ Erreichen persönlicher Ziele (z.B. besser Treppen steigen zu können, mehr Selbständigkeit im Alltag)

□ Mehr Energie im Alltag

□ Spass

□ Körperliche Aktivität gehört zur Routine/Gewohnheit

□ Unterstützung durch andere Personen/Familie

□ Empfehlung durch medizinisches Personal

□ Besser Aussehen

□ Andere: ____________________________

**C5: Was hält Sie persönlich davon ab, körperlich aktiv zu sein?**

0 = überhaupt nicht wichtig – 6 = sehr wichtig

□ Schwindel

□ Kurzatmigkeit

□ Bedenken vor erneuten Infektionen

□ Bedenken vor einer Abstossungsreaktion der Lunge

□ Angst

□ Kein Selbstvertrauen

□ Keine Motivation

□ Müdigkeit

□ Zu viele andere Verpflichtungen/Zeitmangel

□ Zu wenig Energie/Kraft

□ Nebenwirkungen von Medikamenten

□ Begleiterkrankungen

□ Schmerzen

□ Finanzielle Mittel

□ Schlechtes Wetter

□ Keine Möglichkeit, Sport zu treiben

□ Keine Lust, Sport zu treiben

□ Mangel an Wissen, welcher Sport geeignet ist und welche Intensität und Menge erlaubt sind

□ Andere Prioritäten

□ Andere: ______________________________

**C6: Wie würde für Sie ein optimales Trainingsprogramm zur Steigerung der körperlichen Fitness aussehen?**

- *Bitte kreuzen Sie alle zutreffenden Antworten an -*

**Trainingsform:**

□ Krafttraining

□ Ausdauertraining

□ Gleichgewichtstraining

**Trainingsart:**

□ Betreutes Training in einer Gruppe

□ Individuelles Training betreut von einer Physiotherapeut/in

□ Individuelles Training nicht betreut

**Trainingsort:**

□ Zu Hause

□ In der Natur

□ In einem Sportverein

□ In einem Fitnesscenter

□ In einer Institution wie Spital, Rehabilitationszentrum oder Physiotherapie

□ Zuhause mit einer Übungs-App oder einer Übungs-CD/DVD

**Trainingshäufigkeit:**

□ Täglich

□ 1 bis 2 mal pro Woche

□ 3 bis 4 mal pro Woche

□ 5 bis 6 mal pro Woche

**Dauer pro Training:**

□ 10 bis 20 Minuten

□ 20 bis 40 Minuten

□ 40 bis 60 Minuten

□ Mehr als 60 Minuten

□ Kein Training

□ Andere: ________________________________________________________
