# Supplementary material for: Perceptions towards physical activity in adult lung transplant recipients with cystic fibrosis
Source: PLoS One. 2020 Feb 21;15(2):e0229296. doi: 10.1371/journal.pone.0229296 (PMC7034849; doi:10.1371/journal.pone.0229296)
Supplement: S2 Appendix — (DOCX) [file pone.0229296.s002.docx]

**S2 Questionnaire sur l’activité physique des personnes atteintes de fibrose cystique, après transplantation pulmonaire**

*- This questionnaire was translated from the original German version -*

**Langue :**

□ Allemande □ Français

Nous sommes une équipe de recherche conjointe de l'Université de Zurich, de l'Hôpital universitaire de Zurich et de l'Université des sciences appliquées de Zurich. Nous souhaitons en savoir plus sur votre aptitude à l'activité physique et nous interrogeons ici, spécifiquement les personnes atteintes de mucoviscidose (CF) après transplantation pulmonaire, qui sont suivies dans les centres de Bâle, Genève, Lausanne ou de Zurich. Nous avons développé pour ceci, un questionnaire qui en plus des questions médicales, contient essentiellement des questions sur votre aptitude à l’activité physique et sur votre qualité de vie. Ce questionnaire devrait nous aider à en apprendre plus sur vos besoins à ce sujet.

Pour compléter le **questionnaire anonyme**, vous avez besoin d'environ **10-15 minutes**.

**A : Questions générales**

**A1 : Sexe :** □ Féminin □ Masculin

**A2 : Age : _____**

**A3 : Situation familiale :**

□ Pas de partenaire

□ Partenaire en dehors du ménage

□ Partenaire dans le ménage

□ Famille avec des enfants

□ Famille monoparentale

**A4 : Taille (en mètre) :** ______ m (par exemple 1.68m)

**A5 : Poids (en kilogramme) :** ______ kg (par exemple 59.5 kg)

**A6 : Depuis quand avez-vous eu votre transplantation pulmonaire ?**

□ moins de (<) 1 an

□ 1 à < 3 ans

□ 3 à < 5 ans

□ 5 à < 10 ans

□ 10 ans ou plus

**A7 : Avez-vous eu une deuxième transplantation pulmonaire (re-transplantation pulmonaire) ?**

□ Oui □ Non

**A8 : Avez-vous un rejet aigu ou chronique des poumons ?**

□ Oui □ Non

**A9 : Avez-vous d’autres maladies/diagnostics ?**

- Plusieurs réponses possibles -

□ Non

□ Maladie cardiaque

□ Hypertension artérielle

□ Diabète

□ Maladie chronique des reins, dialyse ou transplantation rénale

□ Maladie chronique du foie ou transplantation du foie

□ Cancer de la peau

□ Cancer (sauf cancer de la peau)

□ Dépression

□ Ostéoporose

□ Incontinence

□ Autres : _________________________________________

**A10 : Avez-vous participé à chaque fois à un programme de rééducation après votre(vos) transplantation(s) pulmonaire(s) ?**

□ Oui □ Non

**B : Questions concernant l’éducation/profession**

**B1 : Exercez-vous une activité professionnelle ?**

□ Oui □ Non → Si “Non” passer à la question B3.

**B2 : Quel est le taux de pourcentage de votre activité professionnelle ?** ________%

**B3 : Quelle formation avez-vous faite ou faites-vous en ce moment ?**

- Plusieurs réponses possibles-

□ Université / Haute école spécialisée ou pédagogique / École polytechnique fédérale

□ Maîtrise fédérale, brevet fédéral / École technique ou professionnelle / École professionnelle supérieure / École technique supérieure

□ École de culture générale / Maturité gymnasiale / Maturité professionnelle / École normale ou pédagogique

□ Apprentissage / Formation professionnelle élémentaire

□ École obligatoire achevée

□ École obligatoire inachevée

**B4 : Bénéficiez-vous d’une rente AI ?**

□ Oui □ Non → Si “Non” passer à la question C1.

**B5 : Quel est le pourcentage de votre rente AI ?** __________ %

**C : Questions concernant l’activité physique**

**C1 : Quelle est l'importance de l'activité physique dans votre vie quotidienne ?**

0 = Pas du tout important – 6 = très important

**C2 : Avant la transplantation pulmonaire, combien de fois par semaine (7 jours) avez-vous exercé une activité physique intense ?**

(Ne pas prendre en compte les 2 dernières années avant la transplantation pulmonaire). Des exemples d’activité physique intense : Aérobic, vélo rapide, nager rapidement, sports d’équipe, faire du jogging. En général toutes les activités qui vous font transpirer, respirer rapidement et qui accélèrent votre rythme cardiaque.

Environ____ heure(s) par semaine

**C3 : Comment jugez-vous l’importance de l’activité physique régulière pour votre santé ?**

0 = Pas du tout important – 6 = très important

**C4 : Quelles sont vos motivations personnelles pour être physiquement actif ?**

0 = Pas du tout important – 6 = très important

□ Se sentir mieux

□ Meilleure confiance en soi

□ Amélioration de la qualité de vie

□ Amélioration de la force musculaire

□ Amélioration de l’endurance

□ Contact avec d'autres personnes

□ Réalisation d’objectifs personnels (par exemple mieux monter les escaliers, plus d'autonomie dans la vie quotidienne)

□ Plus d’énergie dans la vie quotidienne

□ Plaisir

□ L'activité physique fait partie de la routine/ de l’habitude

□ Soutien de la famille et d’autres personnes proches

□ Recommandation par du personnel médical

□ Améliorer son apparence

□ Autres : _____________________________________

**C5 : Qu'est-ce qui vous empêche personnellement d'être physiquement actif ?**

0 = Pas du tout important – 6 = très important

□ Vertige

□ Essoufflement

□ Soucis de réinfection

□ Soucis de rejet des poumons

□ Anxiété

□ Pas de confiance en soi

□ Pas de motivation

□ Fatigue

□ Trop nombreux engagements/manque de temps

□ Manque d’énergie/de force

□ Effets secondaires des médicaments

□ Autres maladies

□ Douleurs

□ Ressources financières

□ Mauvais temps

□ Pas de possibilités de faire du sport

□ Pas envie de faire du sport

□ Manque de connaissance quel sport convient et quelle intensité et quantité sont permises

□ D’autres priorités

□ Autres : ____________________________________

**C6 : Comment imaginez-vous un programme d'entraînement optimal pour améliorer votre forme physique ?**

- Veuillez cocher toutes les réponses correspondantes-

**Type d'entraînement :**

□ Entraînement de la force

□ Entraînement d'endurance

□ Entraînement de l'équilibre

**Séances d’entraînement :**

□ Entraînement supervisé en groupe

□ Entraînement individuel supervisé par un physiothérapeute

□ Entraînement individuel non supervise

**Lieu d’entraînement :**

□ À la maison

□ Dans la nature

□ Dans un club sportif

□ Dans un centre de fitness

□ Dans un établissement hospitalier, centre de réadaptation ou physiothérapie

□ À domicile avec une application pratique ou un CD/DVD d'exercices

**Fréquence de l’entraînement :**

□ Tous les jours

□ 1 à 2 fois par semaine

□ 3 à 4 fois par semaine

□ 5 à 6 fois par semaine

**Durée de la séance d’entraînement :**

□ 10 à 20 minutes

□ 20 à 40 minutes

□ 40 à 60 minutes

□ Plus de 60 minutes

□ Pas d’entraînement

□ Autres : _________________________________________________________
