# Supplementary material for: Perceptions towards physical activity in adult lung transplant recipients with cystic fibrosis
Source: PLoS One. 2020 Feb 21;15(2):e0229296. doi: 10.1371/journal.pone.0229296 (PMC7034849; doi:10.1371/journal.pone.0229296)
Supplement: S3 Appendix — (DOCX) [file pone.0229296.s003.docx]

**S3 Questionnaire on perceptions towards physical activity of patients with cystic fibrosis undergoing lung transplantation**

*- This questionnaire was translated from the original German version -*

**Language:**

□ German □ French

We are a Research Group of the University of Zurich, the University Hospital Zurich and the Zurich University of Applied Sciences and are interested to learn more about your perceptions towards physical activity. For this purpose, we specifically interview people with cystic fibrosis (CF) who underwent lung transplantation and currently receive follow-up care in the Centres of Basel, Geneva, Lausanne and Zurich. We have developed a questionnaire that - in addition to medical questions - mainly contains questions on your perceptions towards physical activity and your quality of life. This questionnaire is designed to help us to learn more about your needs on this topic. The next possible step could be the development appropriate training programmes.

You will need about **10-15 minutes** to complete the **anonymous questionnaire**.

**A: General questions**

**A1: Sex:** □ Female □ Male

**A2: Age: _____**

**A3: Marital status:**

□ No partner

□ Partner not sharing household

□ Partner sharing household

□ Family with children

□ Single parent

**A4: Height (in metre):** ______ m (e.g., 1.68m)

**A5: Weight (in kilogramme):** ______ kg (e.g., 59.5 kg)

**A6: How many years ago did you undergo the lung transplantation?**

□ less than (<) 1 year

□ 1 to < 3 years

□ 3 to < 5 years

□ 5 to < 10 years

□ ≥10 years

**A7: Did you undergo a second lung transplant (Re-lung transplantation)?**

□ Yes □ No

**A8: Do you suffer from an acute or chronic lung rejection?**

□ Yes □ No

**A9: Do you have any co-morbidities / other diagnoses?**

- *Multiple responses possible -*

□ No

□ Heart disease

□ High Blood Pressure

□ Diabetes

□ Chronic kidney disease or dialysis or kidney transplantation

□ Chronic liver disease or liver transplantation

□ Skin cancer

□ Cancer (other than skin cancer)

□ Depression

□ Osteoporosis

□ Incontinence

□ Other: _________________________________________

**A10: Have you participated in a rehabilitation programme after lung transplantation?**

□ Yes □ No

**B: Questions regarding your education /job**

**B1: Are you employed?**

□ Yes □ No → If “No” please move on with question B3.

**B2: Job percentage** ________%

**B3: Have you undertaken or are you currently undergoing professional training or an educational course (apprenticeships)?**

- *Multiple responses possible -*

□ University / University of Applied Sciences / University of Teacher Education / ETH

□ Advanced Federal Diploma of Higher Education, Federal Diploma of Higher Education / Technical College / Higher technical college, HTL, HWV

□ Technical secondary school / College / Federal Vocational Baccalaureate / Teaching seminar

□ Apprenticeship

□ Compulsory school completed

□ Compulsory school not completed

**B4: Do you receive a disability allowance?**

□ Yes □ No → If „No“, please move on with question C1.

**B5: What is your percentage of disability allowance?** __________ %

**C: Questions regarding physical activity**

**C1: How important is physical activity for your everyday life?**

0 = not at all relevant – 6 = highly relevant

**C2: How often were you physically active in an average week (7 days) before the transplantation, in the form of vigorous physical activity?**

(Please do not consider the last 2 years before the transplantation). Examples of strenuous physical activity: aerobics, fast cycling, fast swimming, team sports, jogging. In general, all activities where you sweat, your heart rate increases and you need to breathe quickly.

____ day(s) per week (7 days)

**C3: Do you consider regular physical activity as vital to your health?**

0 = not at all relevant – 6 = highly relevant

**C4: What motivates you personally to keep yourself physically active?**

0 = not at all relevant – 6 = highly relevant

□ To feel better

□ Increased self-confidence

□ Improving quality of life

□ Improving muscle strength

□ Improving endurance

□ Social contact

□ To achieve personal goals (e.g., being able to climb stairs more easily, increased daily independence)

□ More energy for everyday life

□ Fun

□ Physical activity is part of (my) routine

□ Support from other persons/family

□ Recommendation by medical staff

□ Better appearance

□ Other: ____________________________

**C5: What keeps you personally from being physically active?**

0 = not at all relevant – 6 = highly relevant

□ Dizziness

□ Shortness of breath

□ Concerns about new infection

□ Concerns about lung rejection

□ Anxiety

□ No self-confidence

□ Lack of motivation

□ Fatigue

□ Too many other commitments/lack of time

□ Low energy/power

□ Side effects of drugs

□ Comorbidities

□ Pain

□ Financial resources

□ Bad weather

□ No possibilities to do physical activities

□ No desire to do physical activities

□ Lack of knowledge regarding recommended exercise

□ Other priorities

□ Other: ______________________________

**C6: What would you consider an optimal training program for yourself in order to increase your physical fitness?**

- *Multiple responses possible -*

**Type of training:**

□ Strength training

□ Endurance training

□ Balance training

**Training supervision:**

□ Supervised training in a group

□ Individual training supervised by a physiotherapist

□ Individual, non-supervised training

**Training venue:**

□ Home-based

□ Outdoors

□ Sports club

□ Fitness centre

□ Institution, e.g. Hospital, Rehabilitation centre or physiotherapy

□ Home-based with exercise-apps or exercise CD/DVD

**Training frequency:**

□ Daily

□ 1 - 2 times per week

□ 3 - 4 times per week

□ 5 - 6 times per week

**Duration per training session:**

□ 10 - 20 minutes

□ 20 - 40 minutes

□ 40 - 60 minutes

□ More than 60 minutes

□ No Training

□ Other: ________________________________________________________
