# Supplementary material for: Perceptions towards physical activity in adult lung transplant recipients with cystic fibrosis
Source: PLoS One. 2020 Feb 21;15(2):e0229296. doi: 10.1371/journal.pone.0229296 (PMC7034849; doi:10.1371/journal.pone.0229296)
Supplement: S4 Appendix — (DOCX) [file pone.0229296.s004.docx]

**S4 Individual responses of survey participants**

- *Translated participant responses -*
- “Aims: gain some weight, no diarrhoea, no dialysis, no pain, no fungal infection and no neuropathic foot pain.”
- “I have a dog that gets me out of doors, besides that I often lack energy to do anything else.”
- “Important for young patients to do some sport! Drink plenty of water, tea or milk.”
- “I consider sports activity to be an important therapy before AND after transplantation! Especially, for physical and psychological balance. After 3 days without sport I get dissatisfied and kind of depressed.”
- “I have never been the sporty type, so the survey was a bit difficult for me to fill out.”
- “Dream: rehabilitation and post-operative support like a professional athlete. The investment would be worthwhile on the long-term course of health!! Take care in the beginning. Fitness, ball sports and personal coaching could help building up deficits in a targeted way. Benefit: long-term body self-confidence = patient will be more active on his own, because PA gets into routine right at the beginning. For the psyche it is important to do sport directly after the transplantation. The time directly after the transplantation is quite exhausting. Sport gets you out of the trauma.”
- “Endurance training is almost impossible, because there is always a physical disability which stops me.”
- “Lack of skills and tremor of the hands.”
- “More medical training therapy or physiotherapy should be paid from health insurance companies per week.”
- “Get more financial support e.g. for sports club fees.”
- “I think that an individual supervised training to improve performance would be a very good thing. It would certainly have a positive effect on psychological well-being as well as on the physical condition.”
